# Yolkin restores cellular immune response and content of T and B cells in lymphoid organs of cyclophosphamide-immunocompromised mice

**DOI:** 10.2478/jvetres-2025-0044

**Published:** 2025-09-06

**Authors:** Michał Zimecki, Jolanta Artym, Maja Kocięba, Ewa Zaczyńska, Angelika Sysak, Marianna Szczypka, Magdalena Lis, Bożena Obmińska-Mrukowicz, Aleksandra Zambrowicz, Łukasz Bobak

**Affiliations:** 1 Laboratory of Immunobiology, Department of Experimental Therapy, Hirszfeld Institute of Immunology and Experimental Therapy, Polish Academy of Sciences, 53-114 Wroclaw, Poland; 2 Department of Pharmacology and Toxicology, Wrocław University of Environmental and Life Sciences, 50-375 Wrocław, Poland; 3 Department of Functional Food Products Development, Wrocław University of Environmental and Life Sciences, 51-630 Wrocław, Poland

**Keywords:** contact sensitivity, humoral response, oxazolone challenge, cyclophosphamide, yolkin, vitellogenin degradation product

## Abstract

**Introduction:**

Yolkin is an egg yolk-derived protein with well-established immunoregulatory activities. In this study its potential restorative properties in cyclophosphamide (CP)-immunocompromised mice were evaluated.

**Material and Methods:**

Mice were treated with a sublethal dose of CP, followed by an access to yolkin contained in drinking water. The mice were immunised with oxazolone (OXA) for contact sensitivity (CS) and with ovalbumin (OVA) for the antibody specific response after 15 or 26 days of treatment with yolkin, respectively. The lymphoid organs were isolated for determination of immune cell numbers and their phenotypes.

**Results:**

A significant suppression of CS following CP treatment and a restoration of this response by yolkin were found. However, no effects of CP and yolkin on the antibody response were registered. In the case of CS a significant loss of T cell numbers in splenocytes was noted. The restoration of CS correlated with reversal of CD3+, CD4+ and CD8+ T cell loss to 88%, 89% and 76.6% of the values observed in the control mice. A significant depletion of the B cells, and all bone marrow cells after CP treatment, was completely restored after 26 day treatment with yolkin.

**Conclusion:**

The presented results add new information to the already described immunoregulatory properties of the protein and indicate a potential of oral yolkin in restoration of cellular immune response in immunocompromised individuals.

## Introduction

One of the challenges of contemporary medicine is associated with preventing the adverse effects of cyclophosphamide (CP) therapy, which is used to treat leukaemia (8), multiple sclerosis (31) or lupus erythematosus (18, 23). Treatment with CP causes immunosuppression because of depletion of cells in bone marrow. Among the immunocompetent cells, rapidly dividing cells like neutrophils are depleted first (9). Recovery of T cells occurs before that of B cells because of their shorter life span (13) and because macrophages are not affected (2). The cytotoxic effects of CP therapy may be ameliorated by applying cytokines, organic compounds and immunotropic proteins. However, the action of the routinely applied recombinant granulocyte colony stimulating factor (filgrastim, a cytokine analogue) is mainly restricted to acceleration of granulopoiesis (11). It is a costly and unstable preparation. Some derivatives of the organic compound isoxazole acted in a directional manner, by promoting renewal of T (36) or B (35, 39) cells in CP-treated mice. Other versatile compounds were also effective in the amelioration of CP-mediated immune suppression (1, 10, 14, 15, 19, 27, 29, 30). These compounds are mostly agonists of Toll-like receptor 4 (TLR4), and their actions encompass the restoration of spleen cell content, cell proliferation, an increase in serum immunoglobulin levels, cytokine production, amelioration of intestinal mucosal injury and improvement of the bacterial intestinal microbiome in CP-immunocompromised mice. Very promising results in CP-induced immune suppression (3, 4, 6) and in a model of CP and busulfan complete stem-cell ablation and cell transplant (5) were obtained with lactoferrin (LF), a protein isolated from milk. Of interest is that LF surpassed filgrastim in renewing immune system function (5).

Studies from the past two decades have revealed another evolutionarily ancient protein contained in the eggs of all oviparous animals. The activity of yolkin, being a degradation product of a large precursor protein of 240 kDa weight from egg yolk – vitellogenin – is associated with a fraction of 35 kDa and a set of smaller low-molecular-weight peptides (24). Recent studies have shown that yolkin is a strong inducer of IL-6, IL-8 and IL-10 cytokines, tumour necrosis factor (TNF) a and interferons (IFNs) in human cells (17, 24, 32, 33) as well as mouse splenocyte cultures (34). Additionally, yolkin has shown several *in vitro* immunomodulatory actions on the production of nitric oxide in a macrophage J744 cell line, lipid peroxidation, viral replication and lipopolysaccharide (LPS)-induced cytokine production (17, 32). Yolkin was also manufactured in a recombinant form and TLR4 was identified as a receptor of it on mouse bone marrow-derived macrophages (16). In these cells, yolkin induced production of nitric oxide, TNF α, IFN a and IFN ß. The induction of cell signalling pathways in these cells was also identified. In studies by the present authors, a stimulatory effect was demonstrated on the humoral immune response to sheep erythrocytes, as was promotion of thymocyte and B cell maturation (22). On the other hand, contact sensitivity (CS) to oxazolone (OXA) was inhibited in adult (22) but not in adolescent mice (37).

The established immunomodulatory characteristics of yolkin, such as amelioration of the immunosuppressive effects of psychic stress in mice (38), and its similarity in function to LF, suggest that it may also counteract CP-induced immune dysfunction. The aim of our study was to explore the effects of oral administration of yolkin on the cellular and humoral immune response of CP-treated mice. In addition to measuring ear oedema thickness in mice with fully developed CS to OXA and detecting specific serum antibodies to ovalbumin (OVA), an analysis of the changes in immune cell number and phenotype in the lymphoid organs was made.

## Material and Methods

### Mice

Eight-week-old female BALB/c mice were purchased from Envigo (Limburg, the Netherlands). They were kept under 12 h/12 h light and dark cycles and given a commercial pelleted feed and filtered tap water *ad libitum*. The Local Ethics Committee for Animal Experiments of the Hirszfeld Institute of Immunology in Wrocław, Poland gave consent under No. 014/2023 for the experiments to be performed. Consideration to the principle of the 3Rs (replacement, reduction and refinement) was given in the experimental design.

### Reagents

Cyclophosphamide (Endoxan) was from Baxter Oncology (Halle, Germany). Hanks’ balanced salt solution (HBSS), phosphate buffered saline (PBS), bovine serum albumin (BSA), 2 M H_2_SO_4_, May–Grünwald–Giemsa stain, Histopaque 1077 1.077 g/mL, incomplete Freund’s adjuvant, oxazolone (OXA) and ovalbumin (OVA) were from Merck (Munich, Germany). Rat anti-mouse CD19^+^ fluorescein isothiocyanate (FITC)/CD3^+^:R-phycoerythin (RPE) (cat. No. DC035), rat anti-mouse CD4^+^:FITC/CD8^+^:RPE (cat. No. DC034), rat anti-mouse CD169:FITC (cat. No. MCA884F) and hamster antimouse CD11c:Alexa488 (cat. No. MCA1369A488) were obtained from Bio-Rad Laboratories (Hercules, CA, USA). Rat anti-mouse CD14:RPE (cat. No. AB-465563) was a product of Invitrogen (Waltham, MA, USA), goat anti-mouse IgG horseradish peroxidase (HRP)-conjugated antibody (cat. No. 1030-05) was supplied by SouthernBiotech (Birmingham, AL, USA) and mouse regulatory T cell (Treg) staining kit #1 is in the eBioscience range (Carlsbad, CA, USA). As a chromogenic substrate, 3,3',5,5'-tetramethylbenzidine (TMB – cat No. 00-4201-56) was used from Bender MedSystems/Thermo Fisher Scientific (Vienna, Austria). Alum adjuvant (Alhydrogel adjuvant 2%) was from InvivoGen (San Diego, CA, USA). Yolkin was isolated from hen eggs and characterised by polyacrylamide gel electrophoresis as previously described (23). The preparation contained two predominant proteins of 40 and 35 kDa with traces of proteins with 30 kDa molecular weight.

### Experimental design

**Fig. 1.**
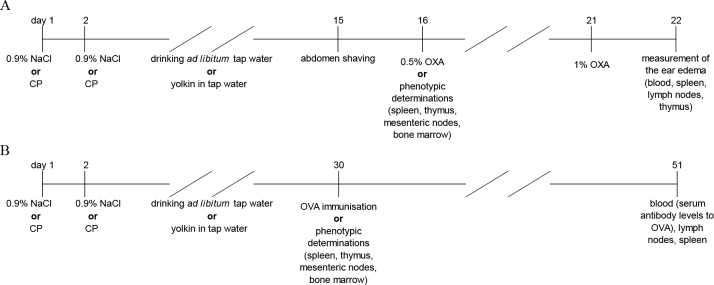
Experimental design. Immunological determinations in (A) the model of contact sensitivity to oxazolone (OXA) and (B) the model of humoral response to ovalbumin (OVA). CP – cyclophosphamide

The experiment was carried out in two models: contact sensitivity (CS) to OXA and humoral response to OVA. In the model of CS to OXA the mice in the experiment were divided into four groups (in each of the group n = 16). The negative control group were injected intraperitoneally (i.p.) with 0.9% NaCl solution in a 0.1 mL volume instead of CP, and drank tap water. The yolkin-only group were likewise injected i.p. with 0.9% NaCl solution instead of CP, and drank tap water with yolkin added to it at 5 μg/mL between days 3 and 15. The CP-only group received CP i.p. in two injections on days 1 and 2 at 50 mg/kg b.w. in a 0.1 mL volume (overall dose 100 mg/kg b.w.), and drank tap water. The CP- and-yolkin group likewise received CP and drank tap water with the same concentration of yolkin for the same period as the yolkin-only group. On day 16 half of each experimental group (n = 8) were anaesthetised and sacrificed by cervical dislocation, and the lymphoid organs were exsected for phenotypic determinations of the immune cells. The rest of each experimental group (n = 8) were immunised with OXA on day 16, the CS reaction was elicited on day 21, and the next day (after 24 h) the ear oedema was measured. The experimental approach is presented in Fig. 1A.

In the model of the humoral response to OVA the mice in the experiment were divided into four groups (in each of the group n = 16). The negative control group were injected i.p. with 0.9% NaCl solution in a 0.1 mL volume instead of CP, and drank tap water. The yolkin-only group were likewise injected i.p. with 0.9% NaCl solution instead of CP, and drank tap water with yolkin added to it at 5 μg/mL between days 3 and 29. The CP-only group received CP i.p. in two injections on days 1 and 2 at 50 mg/kg b.w. in a 0.1 mL volume (overall dose 100 mg/kg b.w.), and drank tap water. The CP-and-yolkin group likewise received CP and drank tap water with the same concentration of yolkin for the same period as the yolkin-only group. On day 30 half of each experimental group (n = 8) were anaesthetised, bled and sacrificed by cervical dislocation, and the lymphoid organs were isolated for phenotypic determinations of the immune cells. The rest of each experimental group (n = 8) were sensitised with OVA on day 30 and after another 21 d (day 51 of the experiment) were anaesthetised, bled and sacrificed by cervical dislocation. The sera were isolated and kept frozen at –80° C until determination of level of anti-OVA IgG antibodies. The experimental approach is presented in Fig. 1B.

### Contact sensitivity to oxazolone

The test was performed according to Noonan and Halliday, with some minor modifications (21). Mice were shaved on the abdomen and 100 μL of 0.5% OXA (the sensitising dose of antigen), dissolved in acetone, was applied to the skin. After 5 d, 50 μL of 1% OXA (the eliciting dose of antigen) was applied to both sides of the auricles. The ear thickness was measured 24 h later by means of spring callipers with an accuracy of 0.05 mm. The results were determined as antigen-specific increases of the ear thickness, *i.e*. baseline measurements were subtracted (these having been measured in the mice given only the eliciting dose of antigen; an additional mouse group/n = 8/was used).

### Humoral immune response to ovalbumin

Ovalbumin was initially dissolved in 0.9% NaCl (0.2 mg/mL) and mixed with the alum adjuvant at 1:1 v/v (50 μL of OVA plus 50 μL of the adjuvant). Mice were immunised subcutaneously in the neck, with 10 μg of OVA (100 μL/mouse) emulsified with the adjuvant at 2%.

### Measurement of serum IgG by reference to a calibration curve

A 96-well plate (Nunc MaxiSorb; ThermoScientific, Roskilde, Denmark) was coated overnight at 4°C with 1 μg/mL OVA in PBS. The plate was then blocked for 2 h at room temperature with a buffer (1% BSA and 0.01% Tween-20 in PBS). A volume of 100 μL of tested sera, diluted 1:500 and 1: 1250 in assay diluent (0.1% BSA and 0.01% Tween-20 in PBS) was added and the plate was incubated for 2 h at room temperature. Each serum sample was tested in duplicate. A series of ten serial dilutions of a positive reference serum was also added to each plate, and each dilution was also tested in duplicate. This positive reference serum was prepared by pooling aliquots from six of the obtained control serum samples that had high antibody titres. The reference serum was arbitrarily defined as containing 1 × 10^3^ units of antibody activity per millilitre. The lowest dilution of the positive reference serum used in the test was 1:1000. Goat anti-mouse IgG HRP-conjugated antibody was added to the wells and incubated at room temperature for 1 h. Between each step and the next, the plate was washed four times (five times after the addition of the HRP-conjugated antibody) using a wash buffer (PBS containing 0.05% Tween 20). A 100 μL volume of TMB substrate solution was added to each well and the plates were incubated at room temperature for 10 min. The reaction was stopped by adding 100 μL of 2M H_2_SO_4_, and absorbance was measured at 450/570 nm using a microplate reader. A reference curve was constructed by plotting the absorbance of each reference sample against its arbitrary antibody units per mL, modelled using a four-parameter logistic fit of the data (Assayfit Pro; AssayCloud, Nijmegen, the Netherlands). Sample antibody units were read off this curve and reported as antibody units/mL.

### Phenotypic determinations of splenocytes, thymocytes, mesenteric lymph node lymphocytes and bone marrow cells

The phenotypic determinations of cells were described in detail in a previous article (23). Briefly, lymphatic organs, including thymuses, spleens, marrow from femurs, and mesenteric lymph nodes, were exsected from euthanised mice. The marrow cavities of the femurs were washed with HBSS to obtain bone marrow cells, while other organs were minced and disaggregated through a nylon mesh into PBS. Splenocytes, thymocytes and mesenteric lymph node cells were centrifuged on a layer of Histopaque 1077 to obtain lymphocytes from the interphase. A trypan blue dye-exclusion assay revealed 90–98% viability of cell suspensions. The lymphocytes from the thymus, spleen and mesenteric lymph nodes (at a concentration of 1 × 10^7^ cells/mL) were stained with CD4^+^:FITC/CD8^+^:RPE dual-colour reagent. The splenocytes, bone marrow cells and lymphocytes from mesenteric lymph nodes were also stained with CD19^+^:FITC/CD3^+^:RPE dual-colour reagent, and splenocytes were additionally stained with rat anti-mouse CD14:RPE and rat anti-mouse CD169:FITC monoclonal antibodies. Hamster anti-mouse CD11c:Alexa488 antibody was also used to stain bone marrow cells. All antibodies were used at the dilutions recommended by the manufacturer. Cells were analysed using flow cytometry.

Percentages of Tregs in splenocytes were evaluated using the commercial mouse regulatory T cell staining kit # 1 following the manufacturer’s instructions. Cells were analysed using flow cytometry, identifying Tregs based on CD25 and Foxp3 expression levels within the CD4^+^ lymphocyte subset (37).

### Statistics

A Brown–Forsythe test was used to determine the homogeneity of variance between groups. When the variance was homogenous, a one-way analysis of variance was applied, followed by *post-hoc* comparisons with Bonferroni or Tukey’s tests to evaluate the significance of the difference between groups. Nonparametric data were evaluated with Kruskal–Wallis analysis of variance. The results are presented as mean values ± standard error of the mean. Significance was determined at P-value < 0.05. The data were analysed statistically using Statistica for Windows software (version 13.3; Dell, Round Rock, TX, USA).

## Results

### Effects of treatment of mice with CP and yolkin in the experimental period on body weight

**Fig. 2.**
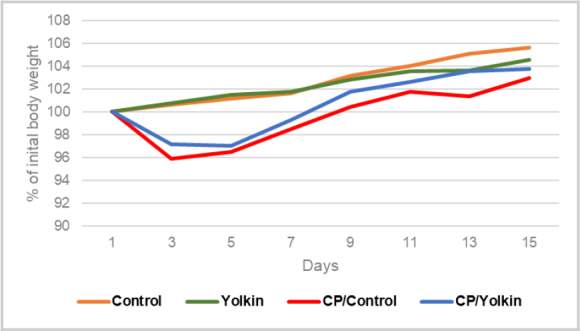
Changes in cyclophosphamide (CP)-immunocompromised mouse body weight during the experimental period. The results are presented as % of initial body weight (mean values) of mice from individual experimental groups from both research models: contact sensitivity to oxazolone and humoral response to ovalbumin. Control – control mice given only 0.9% NaCl; Yolkin – mice given 0.9% NaCl and yolkin; CP/Control – mice given only CP; CP/Yolkin – mice given CP and yolkin

Changes in body weight of the experimental mice, presented as percentages of initial body weight, are shown in Fig. 2. The measurements revealed about a 5% loss of body weight in CP-treated mice 3–9 d following CP administration. The mice that were additionally administered yolkin in their drinking water lost less body weight, particularly during the period of 13–15-d post treatment.

### Effects of oral yolkin administration on contact sensitivity in CP-treated mice

**Fig. 3.**
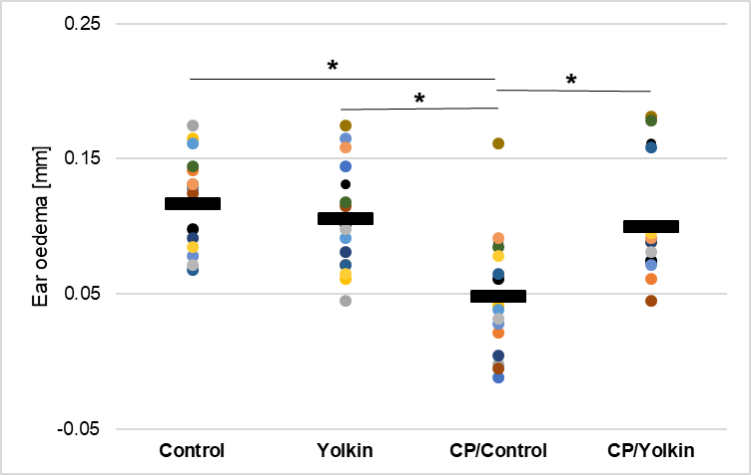
Antigen specific increase in ear oedema in cyclophosphamide (CP)-immunocompromised mice expressing contact sensitivity to oxazolone. The antigen-specific increase of ear thickness (the difference obtained by subtracting the baseline measurement from the measurement taken from sensitised mice) is presented as the mean value of ear thickness measured in eight mice (16 determinations) in mm ± standard error of the mean. * – statistically significant (P-value < 0.05); Control – control mice given only 0.9% NaCl; Yolkin – mice given 0.9% NaCl and yolkin; CP/Control – mice given only CP; CP/Yolkin – mice given CP and yolkin

The treatment of mice with CP significantly inhibited the effectual phase of CS, as measured by the auricle thickness (Fig. 3). Treatment with yolkin alone led to a non-significant decrease in this parameter. The application of yolkin to drinking water for 13 d after administration of CP restored the CS response to 85.5% of the control values.

### Effects of oral yolkin administration for 13 d on total cell numbers and numbers of respective cell phenotypes in the lymphoid organs in CP-treated mice

Table 1 summarises the changes in the total number of cells, cell types and cell subpopulations in the lymphoid organs of mice subjected to CP treatment and exposure to yolkin for 13 d in drinking water.

**Table 1.** Effects of treatment with yolkin for 13 d on total number of immune cells and the numbers of lymphocyte subpopulations in the lymphoid organs of cyclophosphamide (CP)-immunocompromised mice

	Total number of cells in the examined organs					
	Thymus	Spleen	Lymph nodes	Bone marrow		
Control	64.3 ± 4.9 ^ab^	82.1 ± 10.7 ^a^	36.1 ± 2.6 ^a^	21.5 ± 1.3 ^a^		
Yolkin	68.8 ± 5.4 ^a^	87.4 ± 12.9 ^a^	39.1 ± 2.5 ^a^	24.3 ± 2.4 ^a^		
CP/Control	48.7 ± 2.9 ^bc^	61.6 ± 7.7 ^a^	14.8 ± 1.7 ^b^	14.7 ± 1.4 ^b^		
CP/Yolkin	60.0 ± 5.8 ^abc^	75.8 ± 11.6 ^a^	16.1 ± 0.8^b^	14.9 ± 0.9 ^b^		
Thymocytes						
	CD8^+^	CD4^+^CD8^+^	CD4^–^CD8^–^	CD4^+^		
Control	2.9 ± 0.6 ^a^	41.6 ± 3.4 ^a^	2.3 ± 0.2 ^ab^	17.5 ± 1.7 ^a^		
Yolkin	2.9 ± 0.6 ^a^	46.1 ± 4.2 ^a^	2.7 ± 0.2 ^a^	17.2 ± 1.6 ^a^		
CP/Control	1.5 ± 0.2 ^a^	30.1 ± 4.5 ^a^	1.4 ± 0.2 ^c^	10.0 ± 2.1 ^a^		
CP/Yolkin	1.8 ±0.4 ^a^	36.7 ± 5.8 ^a^	1.5 ± 0.3 ^bc^	15.4 ± 2.7 ^a^		
Splenocytes						
	CD8^+^	CD4^+^	CD3^+^	CD19^+^	CD14+CD169^+^	CD25^+^Foxp3^+^
Control	7.7 ± 1.7 ^a^	15.25 ± 1.7 ^a^	28.4 ± 3.9 ^a^	44.6 ± 5.7 ^a^	1.9 ± 0.2 ^a^	6.9 ± 1.0 ^a^
Yolkin	6.7 ± 1.8 ^a^	15.41 ± 3.0 ^a^	29.8 ± 5.7 ^a^	48.0 ± 6.0 ^a^	2.01 ± 0.2 ^a^	7.7 ± 0.9^a^
CP/Control	3.3 ± 0.6 ^a^	7.8 ± 1.3 ^a^	19.1 ± 1.4 ^a^	33.7 ± 5.2 ^a^	2.7 ± 0.5 ^a^	6.1 ± 0.7 ^a^
CP/Yolkin	5.9 ± 1.1 ^a^	13.6 ± 2.4 ^a^	25.5 ± 3.2 ^a^	38.7 ± 6.8 ^a^	3.1 ± 0.6 ^a^	6.2 ± 1.2 ^a^
Mesenteric lymph node cells						
	CD8^+^	CD4^+^	CD3^+^	CD19^+^		
Control	5.4 ± 0.6 ^a^	21.1 ± 1.8 ^a^	25.3 ± 2.1 ^a^	10.2 ± 0.6 ^a^		
Yolkin	4.6 ± 0.1^a^	21.1 ± 1.6 ^a^	25. 9 ± 1.8 ^a^	9.2 ± 1.5^a^		
CP/Control	2.1 ± 0.2 ^b^	9.9 ± 1.1 ^b^	12.0 ± 1.4 ^b^	2.3 ± 0.3^b^		
CP/Yolkin	2.6 ± 0.1 ^b^	10.4 ± 0.5 ^b^	12.7 ± 0.5 ^b^	3.1 ± 0.3^b^		
Bone marrow cells						
	CD19^+^					
Control	12.8 ± 0.9 ^ab^					
Yolkin	14.5 ± 1.4 ^a^					
CP/Control	9.7 ± 0.8 ^bc^					
CP/Yolkin	9.0 ± 0.9 ^bc^					

1 The results are presented as the number of cells (×10^6^) of appropriate lymphocyte subpopulations (mean ± standard error of the mean); values with different letters in the column differ significantly (P-value < 0.05)

The total cell numbers in the investigated organs decreased in all organs, but statistically significant decreases occurred only in the lymph nodes and bone marrow. The cell loss in these organs was not reversed in mice ingesting yolkin. The decreases in respective T cell subpopulations also took place in the thymus, although a significant decrease occurred only in the CD4^–^CD8^–^ subset, the level of which was not changed by yolkin. The losses of T cell subsets and CD19^+^ B cells were quite marked in the spleen after CP treatment. Although not statistically significant, they were associated with a reversal of CD3^+^, CD4^+^ and CD8^+^ T cell loss to respective 88%, 89% and 76.6% of the values observed in the untreated control group. These changes did not extend to the levels of macrophages in the spleen. A decline of cell numbers was also evident in the mesenteric lymph nodes, and B cells were particularly affected. No reconstitution of the cell numbers by yolkin was observed. In addition, no changes in the B cell numbers in the bone marrow nor in T regulatory CD25^+^Foxp3^+^ cells in the spleen were found.

### Effects of yolkin administration for 26 d on the humoral immune response to ovalbumin in CP-treated mice

**Fig. 4.**
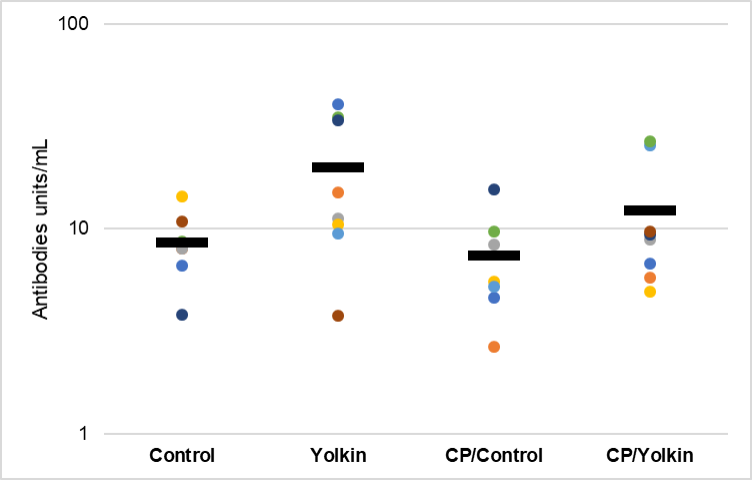
The levels of antibodies to ovalbumin in cyclophosphamide (CP)-immunocompromised mice followed by 26-d oral treatment with yolkin. The data are presented as mean and individual values of the antibody levels on a logarithmic scale. Control – control mice given only 0.9% NaCl; Yolkin – mice given 0.9% NaCl and yolkin; CP/Control – mice given only CP; CP/Yolkin – mice given CP and yolkin

The results show that the antibody levels were enhanced to some degree by the treatment with yolkin on its own and slightly diminished by CP treatment (Fig. 4). The treatment of CP-immunocompromised mice with yolkin showed only a tendency to not significantly elevate the serum antibody concentration above the value noted in the untreated control mice. In general, the registered changes in the antibody levels were minor.

### Effects of oral yolkin administration for 26 d on total cell numbers and numbers of respective cell phenotypes in the lymphoid organs in CP-treated mice

The total cell numbers in the thymus, expressed as percentages, significantly decreased after treatment of mice with CP (Table 2). Although yolkin did not increase the cell numbers in a significant manner after CP treatment, numbers did not significantly differ from those observed in untreated control mice. The total number of splenocytes significantly decreased (by 41.9%) after CP treatment and did not recover after administration of yolkin. However, in the case of mesenteric lymph nodes, a significant decrease in the cell content was partially restored by yolkin and these cell numbers were not significantly different from the values registered in the untreated control mice. Notably, the most pronounced changes with regard to the effect of CP and yolkin were noted in the bone marrow, where a very significant depletion of cell numbers mediated by CP was completely restored by the yolkin treatment.

**Table 2.** Effects of treatment with yolkin for 26 d on the total number of cells and the numbers of lymphocyte subpopulations in the lymphoid organs of cyclophosphamide (CP)-immunocompromised mice

	Total number of cells in the examined organs					
	Thymus	Spleen	Lymph nodes	Bone marrow		
Control	97.3 ± 3.5 ^a^	141.2 ± 6.7 ^a^	34.05 ± 1.4 a^b^	20.3 ± 1.0 a^b^		
Yolkin	94.3 ± 2.8 ^ab^	126.9 ± 3.7 ^a^	37.5 ± 2.9 ^a^	21.1 ± 1.2 ^a^		
CP/Control	82.1 ± 3.1 ^bc^	82.0 ± 6.2 ^b^	24.7 ± 1.9 ^c^	11.6 ± 0.8 ^c^		
CP/Yolkin	93.0 ± 4.5 ^abc^	92.9 ± 5.2 ^b^	30.6 ± 2.6 ^abc^	20.2 ± 2.0 ^ab^		
Thymocytes						
	CD8^+^	CD4^+^CD8^+^	CD4^–^CD8^–^	CD4^+^		
Control	3.9 ± 0.4 ^a^	59.2 ± 4.2 ^a^	7.3 ± 1.4 ^a^	25.9 ± 1.8 ^a^		
Yolkin	4.1 ± 0.4 ^a^	54.9 ± 2.7 ^ab^	6.0 ± 0.5 ^a^	28.6 ± 3.3 ^a^		
CP/Control	4.1 ± 0.5 ^a^	43.0 ± 2.3 ^c^	5.1 ± 0.5 ^a^	30.1 ± 1.5 ^a^		
CP/Yolkin	4.9 ± 0.7 ^a^	50.4 ± 1.8 ^abc^	5.1 ± 0.5 ^a^	32.6 ± 2.2 ^a^		
Splenocytes						
	CD8^+^	CD4^+^	CD3^+^	CD19^+^	CD14^+^CD169^+^	CD25^+^Foxp3^+^
Control	12.6 ± 0.6 ^a^	24.5 ± 1.0 ^a^	48.6 ± 2.8 ^a^	76.8 ± 3.3 ^a^	3.4 ± 0.4 ^a^	11.4 ± 0.8 ^a^
Yolkin	8.1 ± 0.4 ^b^	20.5 ± 1.1 ^ab^	38.7 ± 1.2 ^b^	75.3 ± 3.4 ^a^	4.5 ± 0.8 ^a^	10.4 ± 0.5 ^a^
CP/Control	4.4 ± 0.8 ^c^	14.2 ± 1.7 ^c^	25.4 ± 3.3 ^c^	44.4 ± 2.4 ^b^	3.1 ± 0.4 ^a^	6.6 ± 0.5 ^b^
CP/Yolkin	7.3 ± 0.9 ^b^	15.4 ± 1.1 ^bc^	31.1 ± 2.2 ^bc^	52.0 ± 3.4 ^b^	4.0 ± 0.8 ^a^	7.2 ± 0.7 ^b^
Mesenteric lymph node cells						
	CD8^+^	CD4^+^	CD3^+^	CD19^+^		
Control	4.2 ± 0.4 ^a^	19.6 ± 0.7 ^a^	22.5 ± 1.6 ^a^	9.9 ± 1.3 ^a^		
Yolkin	4.2 ± 0.3 ^a^	19.7 ± 1.3 ^a^	23.0 ± 1.2 ^a^	13.2 ± 2.1 ^a^		
CP/Control	3.3 ± 0.4 ^a^	17.4 ± 1.5 ^a^	20.6 ±.1.8 ^a^	3.2 ± 0.4 ^b^		
CP/Yolkin	4.0 ± 0.4 ^a^	21.6 ± 1.7 ^a^	24.9 ± 2.1 ^a^	4.0 ± 0.7 ^b^		
Bone marrow cells						
	CD19^+^					
Control	13.8 ± 0.6 ^a^					
Yolkin	12.6 ± 0.8 ^ab^					
CP/Control	7.7 ± 0.5 ^c^					
CP/Yolkin	13.1 ± 0.6 ^ab^					

1 The results are presented as the number of cells (×10^6^) of the appropriate lymphocyte subpopulations (mean ± standard error of the mean); values with different letters in the column differ significantly (P-value < 0.05)

The analysis of changes in cell numbers of respective cell phenotypes in the lymphoid organs showed that the CD4^+^CD8^+^ cell subset of thymocytes was significantly reduced and partially recovered following yolkin treatment. In the spleen, there were significant decreases in the T cells of the CD3^+^, CD8^+^ and CD4^+^ phenotypes after CP treatment and CD19^+^ B cells. In the case of CD8^+^ cells, yolkin significantly restored the cell level registered in CP-treated mice. Of interest is that yolkin alone lowered CD8^+^ T cell number. In addition, the number of splenic regulatory T cells (CD25^+^Foxp3^+^) was significantly reduced but was not restored by yolkin. The level of macrophages (CD14^+^CD169^+^) was not affected. In the mesenteric lymph nodes, a strong decrease in the CD19^+^ B cell number after CP treatment was found, but was not affected by yolkin treatment. Importantly, CD19^+^ B cells in the bone marrow were deeply depleted by CP, but were replenished by yolkin to the control level registered in untreated control mice.

## Discussion

In this study we showed that yolkin administered in drinking water following CP treatment significantly reversed CP-suppressed CS to OXA in BALB/c mice, and partially or completely restored cell contents in the lymphoid organs. On the other hand, the humoral immune response to OVA was neither significantly affected by CP nor modified by the yolkin treatment. The treatment of CP-immunocompromised mice with yolkin also correlated with a better body weight gain in these mice. The renewal of cell content in the lymphoid organs was more significant following the more prolonged administration of yolkin. Nevertheless, the changes in the total cell content and the proportions of respective T cell phenotypes after 13-d treatment with yolkin may partially explain the stimulatory action of yolkin on CS in CP-immunosuppressed mice.

That the number of B cells in the lymphoid organs was not restored was probably because their life spans are much longer than those of T cells (13). However, among the splenocytes, a quite meaningful restoration of T cell subsets was found. Effector T cells are needed for the development of CS, although a primary role for initiation of CS is ascribed to B-1 B cells (7). Although no reports are available with regard to their resistance to CP, this long-lived, nonproliferating B cell subset (20) was not affected by CP action. Thus, the reconstitution of CS by yolkin may result from its effects of increasing recruitment of T cells in the spleen (Table 1) and promoting body weight gain (Fig. 2). Beneficial effects of yolkin on the mucosal integrity and gut bacterial composition cannot be excluded, and these would represent benefits to the overall health status of the mice. Such effects of compounds in the reconstitution of the immune response in CP-treated mice were reported (14, 15, 19, 29). In addition, the roles of IFN γ, IL-6 and TNF α in the development of CS (12) in immunocompromised mice must not be underestimated, since yolkin was shown to be a strong inducer of these cytokines in *in vitro* (17, 25, 33, 34) and *ex vivo* experiments (38).

After 13 d of CP treatment, a serious loss of B cell content in the bone marrow was observed, which was not restored by yolkin, just as CD4^–^CD8^–^ thymocyte immature cell subsets were not. These cells in the primary lymphoid organs quickly divide, and are thus highly susceptible to CP treatment. Nevertheless, after 26 d of treatment with yolkin, the even higher B cell loss in the bone marrow was fully reconstituted by yolkin. The results indicate that depletion of B cells in the bone marrow requires a prolonged period of yolkin administration. In general, the depletion of CD19^+^ B cells by CP in the lymphoid organs was delayed and more significant 26 d after CP administration. The total cell number in the mesenteric lymph nodes measured on day 15 following CP treatment fell by 52.5%. This decrease was probably predominantly associated with B cell depletion (by 77.5%). However, after 26 d of yolkin administration, the effect of CP injection was mostly eliminated, since the cell content in the lymph nodes attained 89.9% of the level registered in nonmanipulated control mice. On the other hand, the severely diminished T cell levels in the spleen 15 d after CP administration substantially recovered after treatment with yolkin. This CP-mediated effect was not observed after 26-d treatment. It seems that following this prolonged period after the application of the immunosuppressive agent, the T cell reservoir underwent a natural recovery because of the short life span of this cell type. No significant changes were observed in CP-treated mice in the content of macrophages or dendritic cells (not shown) in the bone marrow. Additionally, the treatment of mice with CP did not affect the level of T regulatory cells, although the content of this cell subset played a role in the interpretation of the stimulatory effects of yolkin on CS in juvenile mice (37).

In the present experimental protocol using a long-term treatment of mice with yolkin, the protein exerted no stimulatory effect, and we did not note a significant decrease of the antibody level following CP despite decreased levels of helper type CD4^+^ and CD19^+^ cells in the spleen upon immunisation with OVA. In fact, these lower cell numbers were not recovered by yolkin. Nevertheless, the observed minor changes in the antibody levels were anticipated changes in the respective mouse groups. It is possible that a more optimal experimental protocol could have revealed a significant restorative effect of yolkin on the humoral immune response in CP-treated mice.

We managed to demonstrate a reconstituting effect on the humoral immune response in CP-treated mice by LF, another potent immune regulator and agonist of TLR4 (25). In this model, orally administered LF restored the humoral immune response, but only to some degree (5, 6). Nevertheless, the models using yolkin and LF differed in terms of the experimental design and the antigens used (sheep red blood cells in the case of LF). Additionally, the number of antibody-forming cells in the spleen was measured, and not the antibody titre.

We also envisage that yolkin may induce myelopoiesis like LF, which accelerates the renewal of granulopoiesis through its ability to induce GSF (28). Such a mechanism is very likely in the case of yolkin, as it is a strong TLR agonist. In fact, the level of blood neutrophils in mice treated with yolkin and in which CS developed was higher than in control animals (37). In addition (data to be published), mice that ingested yolkin prior to LPS injection had enhanced levels of granulocytes in the blood. Thus, beside lymphopoiesis, yolkin may also induce myelopoiesis. Such a property may predispose yolkin to use as a preventive measure against bacterial infection.

In summary, this study showed that yolkin administered orally in drinking water significantly recovered CP-induced immunosuppression of contact sensitivity in BALB/c mice. This effect correlated with the renewal of T cell content in the spleen. Although neither marked suppression nor reconstitution of the antibody response was found in this model, the prolonged treatment of CP-immunocompromised mice with yolkin led to a complete reconstitution of the total cell and B cell numbers in the bone marrow.

## Conclusion

To conclude, evidence is accumulating that yolkin, which under normal conditions suppresses cellular immune response in adult mice, exhibits immunostimulatory or normalising effects on the altered parameters of the immune system in immature or experimentally manipulated mice (stressed or immunocompromised animals). In addition, taking into account its protective effects on the central nervous system, it may be concluded that certain egg-derived proteins contained in nutraceuticals, as well as the consumption of eggs in normal diets (26), may offer benefits for human health.
